# ERβ Isoforms Have Differential Clinical Significance in Breast Cancer Subtypes and Subgroups

**DOI:** 10.3390/cimb44040107

**Published:** 2022-04-06

**Authors:** Young Choi, Hadong Kim, Simcha Pollack

**Affiliations:** 1 Department of Pathology, Yale School of Medicine, 310 Cedar Street, New Haven, CT 06510, USA; 2 Research Institute 21, Dong-A ST, Geumhwa-ro, 105 Beon-gil, Giheung-gu, Yongin 17073, Korea; hadd1318@naver.com; 3 Department of Statistics, St. John’s University, Queens, NY 11423, USA; simcha.pollack@stjohns.edu

**Keywords:** breast cancer, estrogen receptor beta, outcome assessment, survival analysis, prognosis, therapeutic use

## Abstract

**Simple Summary:**

ERβ, an ER subtype first identified in 1996, is significantly expressed in ERα-negative breast cancer (BCa) and TNBC. Many studies investigated mostly ERβ1 protein expression in the entire cohort of BCa, and the results are inconsistent. In this study, we simultaneously investigated both ERβ mRNA and three ERβ 1, 2, and 5 protein isoforms in various subtypes and subgroups of BCa. Each ERβ isoform’s mRNA and protein expression seemingly plays a significant role in BCa subtypes and subgroups, and ERβ2 mRNA expression is risk factor for poor outcome. Studies in a large cohort of BCa are needed to explore the potential usefulness of ERβ as a prognostic and predictive marker and a therapeutic target in BCa. Furthermore, the standardization of a ERβ testing protocol may be required for ERβ testing to be utilized in a clinical setting.

**Abstract:**

ERβ, an ER subtype first identified in 1996, is highly expressed in different types of BCa including ERα-negative BCa and TNBC. Many studies on ERβ expression investigated mostly on ERβ1 protein expression in ERα-positive and ERα-negative BCa combined. The results are conflicting. This may be due to the complexity of ERβ isoforms, subject heterogeneity, and various study designs targeting different ERβ isoforms and either ERβ protein or mRNA expression, as well as to the lack of a standardized testing protocol. Herein, we simultaneously investigated both mRNA and protein expression of ERβ isoforms 1, 2, and 5 in different BCa subtypes and clinical characteristics. Patient samples (138) and breast cancer cell lines (BCC) reflecting different types of BCa were tested for ERα and ERβ mRNA expression using quantitative real-time PCR, as well as for protein expression of ERα, ERβ1, ERβ2, and ERβ5 isoforms, PR, HER2/neu, Ki-67, CK 5/6, and p53 using immunohistochemistry. Associations of ERβ isoform expression with clinical characteristics and overall survival (OS) were analyzed. ERβ1, 2, and 5 isoforms are differentially expressed in different BCa subtypes including ERα-negative and TNBC. Each ERβ isoform seemingly plays a distinct role and is associated with clinical tumor characteristics and patient outcomes. ERβ isoform expression is significantly associated with >15% Ki-67 positivity and poor prognostic markers, and it predicts poorer OS, mostly in the subgroups. High ERβ2 and 5 isoform expression in ERα-negative BCa and TNBC is predictive of poor OS. Further investigation of ERβ isoforms in a larger cohort of BCa subgroups is needed to evaluate the role of ERβ for the potential usefulness of ERβ as a prognostic and predictive marker and for therapeutic use. The inconsistent outcomes of ERβ isoform mRNA or protein expression in many studies suggest that the standardization of ERβ testing would facilitate the use of ERβ in a clinical setting.

## 1. Introduction

### Two Estrogen Receptors

There are two estrogen receptor (ER) genes (*ESR1/ERα and ESR2/ERβ*). ERα and ERβ are members of the nuclear receptor superfamily of transcription factors and share some structural similarities including a high degree of homology (96%) in their DNA-binding regions. However, they also have distinct differences in genotype, tissue distribution, and binding to pharmacological agents; they share only moderate homology in the ligand-binding region, and they have markedly distinct NH^2^-terminal activation function-1 (AP-1) regions. ERα and ERβ can form heterodimers [1]; when co-expressed, ERβ acts as a trans-dominant inhibitor of ERα transcriptional activity. Thus, the relative levels of ERα and ERβ in BCa are likely to impact cell proliferation, signaling pathways, and their response to ER ligands [2,3].

*ESR2* can encode several different ERβ isoforms owing to exon deletions or alternative splicing of the last coding exon (exon 8) truncated at the C-terminus. Full-length ERβ1 is the primary ERβ isoform that mediates gene expression and response to estrogen or ERβ-selective ligands, and it is an obligatory partner in ERβ dimers, whereas the other isoforms function as variable dimer partners [4]. However, ERβ2/βcx preferentially forms a heterodimer with ERα rather than with other ERβ isoforms, and it shows a significant dominant negative activity against ERα transactivation [5,6]. ERβ5 isoform has also been shown to have estrogen–independent transcriptional properties, and this could contribute to the significant role of ERβ5 in BCa [7]. Thus, each ERβ isoform could play a significant role in BCa.

Modern molecular-based diagnostic tools have elucidated the phenotypic and molecular heterogeneity of breast cancer (BCa) [8], including luminal A, luminal B, basal-like, and human epidermal growth factor receptor 2 (HER2) types. The current primary treatment for ERα-positive BCa is endocrine therapy with selective ER modulators and an aromatase inhibitor based on positive ERα nuclear expression. However, ERα is overexpressed in 60–70% of BCa, and de novo resistance to estrogen modulators is exhibited in approximately 50% of cases [9]. ERα-negative tumors comprise 30% of all BCa, and triple-negative BCa (TNBC), which lacks ERα, PR, and HER2 and accounts for 5–20% of all BCa, has poor outcomes and an aggressive clinical behavior [10]. The current treatment for ERα-positive breast cancers is endocrine therapy based on positive ERα nuclear expression [9]. ERα-negative BCa and TNBC do not get the benefit of endocrine therapy, and only 20% of TNBCs respond to standard chemotherapy. Thus, novel treatments are needed for treating ERα-negative BCa and TNBC.

ERβ, an ER subtype first identified in 1996 [11], is significantly expressed in various types of BCa including ERα-negative BCa and TNBC. Many studies have investigated the potential usefulness of ERβ as a prognostic and predictive marker and as a therapeutic target in BCa. A large portion of previous ERβ studies in BCa investigated ERβ1 protein expression, although some examined ERβ mRNA, in ERα-positive and/or ERα-negative BCa and TNBC [12,13,14,15,16,17]. The results were inconsistent; ERβ mRNA or protein expression of ERβ isoforms in various cohorts were shown to be associated with different clinical outcomes, both favorable and adverse. In ERα-negative and TNBC, ERβ expression was shown to be associated with high Ki-67 positivity, implicated in the estrogen-independent growth of BCa, with either favorable or adverse outcomes. Thus, an accurate determination of ERβ expression in ERα-negative BCa and TNBC could provide a basis to treat a large number of women with safe and effective hormonal-based therapies which at present are not considered as an option in this cohort of patients [17,18,19].

In a previous study [20], we found that high ERβ1 protein expression in ERα-negative BCa was correlated with high Ki-67, P53, and Her2/neu expression. In this study, we simultaneously investigated both mRNA and protein expression and we tested ERβ1, 2, and 5 isoforms in the entire cohort, as well as in various subtypes and subgroups, as the use of a single ERβ isoform is unlikely to reveal the complete biological significance of total ERβ isoform expression in BCa. The mRNA or protein expression of each ERβ isoform was correlated with various clinical characteristics and clinical outcomes.

## 2. Materials and Methods

### 2.1. Patients

All procedures involving study subjects were performed in accordance with the ethical standards of the Institutional Research Board, The Bridgeport Hospital, Bridgeport, CT (IRB# 090101). The study comprised 65 ERα-negative (43 TNBC) and 73 ERα-positive subjects in a total of 138 BCa patients with a follow-up period from 2003 to 2010. Demographic and clinical characteristics of all subjects were retrieved from medical records and cancer registry reports, as well as pathology records for hormone receptor reports, histologic types, tumor size, and AJCC tumor stages. The BCa histologic types included 109 infiltrating duct carcinoma NOS, seven atypical medullary, six medullary, three apocrine, three infiltrating lobular, two inflammatory, five mixed ductal/lobular, and three micropapillary types. Histological grades were assessed according to the Bloom–Richardson classification criteria. The AJCC tumor stages consisted of 75 stage I, 45 stage II, and 18 stage III. For treatment, nine underwent surgery only, 10 received hormonal therapy only, 42 underwent radiation followed by hormonal therapy, and 77 had chemoradiotherapy. The follow-up period ranged from 1 to 96 months (median, 60 months); 20 patients died during this period. The phenotypic BCa patterns were determined according to ERα, HER2/neu, and progesterone receptor (PR) status following consensus guidelines. The proliferation marker Ki-67) was evaluated for all tumors. The molecular types comprised 50 luminal A (ERα^+^/PR^+^/HER2^−^), 25 luminal B (ERα^+^ and/or PR^+^/HER2^+^/Ki-67^+^), 17 HER2 type (ERα^−^/PR^−^/HER2^+^), 17 basal-like type (ERα^−^/PR^−^/HER2^−^/CK5/6^+^), and 29 unclassified [8]. 

### 2.2. Breast Cancer Cell Lines 

Multiple BCC lines reflecting a range of BCa phenotypes and molecular types [21] were tested as control for ERβ expression assessment in different types of BCa, including luminal A type (ZR-75, MCF-7, and T-47D), luminal B type (MDA-MB-361, BT 474), HER2 type (SK-BR3), and basal-like type (MDA-MB-231, BT20, MDA-MB-468, and Hst578). BCCs were either purchased from American Type Culture Collection or kindly gifted by colleagues.

### 2.3. Tissue Microarray (TMA) Preparation

Hematoxylin and eosin sections of formalin-fixed paraffin-embedded (FFPE) tumor samples were evaluated. TMA blocks were constructed using triplicate 0.6 mm diameter cores selected from the most representative tumor cellular areas of the primary BCa and the FFPE block of BCa cell lines. 

### 2.4. RNA Isolation and Quantitative Reverse Transcription (qRT)-PCR of ERα and ERβ Isoforms

Three tumor cores were acquired from the same primary BCa used for TMA and from formalin-fixed cell buttons of BCC. RNA was isolated using the RNeasy RNA isolation kit according to the manufacturer’s protocol (Qiagen, Hilden, Germany). RT-PCR was performed using FastStart Universal SYBR Green master mix (Roche, Basel, Switzerland) and monitored using an Eppendorf Realplex 2.0 (Eppendorf, Framingham, MA, USA). The RNA integrity of tumor tissues and BCC was verified via electrophoretic separation on 1.5% agarose gels and by amplification of the constitutively expressed *ACTB* gene. Expression of ERβ1, ERβ2, and ERβ5 mRNA was tested via conventional qRT-PCR in an automatic thermal cycler (MJ Research, Waltham, MA, USA). The isoform-specific sense and antisense primers were as follows: *ERα*, 5′–TCCTCATCCTCTCCCACATC–3′ and 5′–TCTCCAGCAGCAGGTCATAG–3′ (ref. NM_000125, 1757–1861, 105 bp); *ERβ-1*, 5′–GATGCTTTGGTTTGGGTGAT–3′ and 5′–GGTCATACACTGGGACCACA–3′ (ref. NM_001437, 1771–1936: 166 bp); *ERβ-2*, 5′–TGGCTAACCTCCTGATGCTC–3′ and 5′–TGGATTACAATGATCCCAGAGG–3′ (ref. NM_001040276, 2107–231: 208 bp and NM_001040275, 1832–2039: 208 bp); *ERβ-5′*–GTTTGGGTGATTGCCAAGAG–3′ and 5′–TTGCAGACACTTTTCCCAAA–3′ (ref. DQ838583.1, 1312–1496: 185 bp), and *ACTB*, 5′–GATGAGATTGGCATGGCTTT–3′ and 5’–CACCTTCACCGTTCCAGTTT–3’ (ref. NM_001101, 1276–1375: 100 bp). *ACTB* and hypoxanthine ribosyltransferase (HPRT) were used as control genes to determine RNA integrity and RT efficiency. The PCR reaction mixture consisted of 8 nmol/L each of the forward and reverse primers, 100 nmol/L probe, 125 μmol/L deoxynucleotide triphosphate, and 5 mmol/L MgCl_2_. PCR was performed using Perkin-Elmer 9600 thermal cyclers. The PCR program was 45 cycles at 95 °C for 15 s, and at 60 °C for 45 s. All samples were amplified in triplicate; RT-PCR was repeated for every isoform and normalized to the copy numbers of *ACTB* gene. The comparative Ct method was used to normalize mRNA copy numbers of ERα and ERβ in tumor samples. The absolute quantification of each isoform was compared to a standard graph generated using a serially diluted synthetic reference solution and normalized to *ACTB*. Positive and negative controls of BCa tissues and BCCs were included in each reaction plate. As quality control for RNA integrity in formalin-fixed breast tissue, fresh and formalin-fixed BCCs were tested for ERβ mRNA, and the levels of ERβ mRNA expression were compared in both samples. The cutoff value of ERβ isoform mRNA was determined separating lower- to high-level mRNA values by observing the cutoffs of the corresponding ERβ proteins [22]. A logistic regression analysis for a range of possible cutoffs for the mRNA variable was performed using the cutoffs of ERβ1, ERβ2, and ERβ5 protein expression at 20%, 20%, and 40%, respectively. The threshold maximizing the AUC (area under the receiver operating curve) was chosen. This resulted in mRNA positive cutoff values of 14 × 10^6^, 13 × 10^6^, and 1 × 10^6^ for ERβ1, ERβ2, and ERβ5 mRNA, respectively (SAS 9.4v, Cary, NC, USA).

### 2.5. Immunohistochemistry 

Standard immunohistochemistry (IHC) was performed using 4 µm thick sections of TMA slides of BCa and BCCs following antigen retrieval with a steam-heating (95 °C) system in 0.01 M citrate buffer (pH 6.0) for 20 min or 1 mmol/L Tris-EDTA buffer at pH 9.0. Sections were stained with appropriately diluted antibodies (Table 1) using an automated immunostainer (Dako, Santa Clara, CA, USA). 

The selection of ERβ antibodies used in our study was determined by reviewing previous studies on various ERβ antibodies [23,24,25] and by reviewing the specificity and sensitivity of ERβ antibodies provided in the manufacturer’s data. Different clones of each ERβ isoform antibody were tested for the optimum and reproducible immunoreaction in repeat testing using negative and positive staining and tissue controls, following the standard immunohistochemistry testing protocol established for ERα expression in our laboratory [26].

The immunoreaction of nuclear staining was evaluated using a semiquantitative Allred scoring system [27], summing the proportion of positive cells (scored on a scale of 0–5) and staining intensity (scored on a scale of 0–3) to produce a cumulative score of 8. A total score of 0–2 was regarded as negative, and a total score > 3, with 1–10% weakly positive cells or >20% nuclear positivity, were taken as the cutoffs of positivity for ERβ1 and 2 isoforms, while >40% was applied for ERβ5 protein expression [28,29,30]. Some commercially available ERβ antibodies were either nonspecific or insensitive for the detection of ERβ and exhibited an appreciable level of background, as well as variable nuclear and cytoplasmic staining. Polyclonal antibodies showed more background and cytoplasmic reaction. High levels of cytoplasmic staining were detected by the antibody produced using the N-terminal domain. Upon higher dilution and testing of the antibodies, the background and cytoplasmic reaction were reduced and optimized. As the significance of the cytoplasmic reaction of ERβ antibodies requires thorough characterization with regard to sensitivity and specificity [24,25], the cytoplasmic reaction was not evaluated for clinical outcomes in our study.

Over 1% of ERα and PR nuclear staining was considered positive, but cytoplasmic reaction of ER or PR in BCa was not assessed as in the ASCO/CAP guidelines [26]. HER2/neu expression was interpreted following the HercepTest kit guidelines. HER2 staining was scored according to the ASCO/CAP guidelines and considered positive for 3+ HER2 staining or 2+ HER2 staining with fluorescent in situ hybridization positivity. A nuclear immune reaction of Ki-67 > 15% with p53 > 5% and strong cytoplasmic staining of CK5/6 was considered positive.

### 2.6. Statistical Analysis

The associations between ERβ isoform protein and mRNA expression and clinical characteristics were assessed for the entire cohort and subtypes and subgroups of BCa by Fisher’s exact test. The frequency of each ERβ isoform expression in subtypes was assessed by McNemar’s test, while the correlation between ERβ isoform expression and clinical characteristics was assessed by Spearman’s rank-order test. Overall survival (OS) was calculated from the date of BCa diagnosis to that of death or the last follow-up visit, and OS outcomes were estimated using Kaplan–Meier (KM) curves for censored data using the log-rank tests and using Cox univariate and multivariate proportional hazard (PH) regression models. Hazard ratios were determined with 95% confidence intervals. A *p*-value < 0.05 was defined as significant (SAS 9.4v, SAS Institute Inc, Cary, NC, USA). 

## 3. Results 

### 3.1. Differential mRNA and Protein Expression of ERα and ERβ Isoforms in Benign Breast Tissues and BCa Subtypes

ERβ mRNA expression levels in BCa were lower than those in benign breast tissue, and those of ERα expression. ERβ1 mRNA in BCa ranged from 1.0 to 6000 × 10^6^, ERβ2 ranged from 1.0 to 51,000 × 10^6^, and ERβ5 ranged from 1.0 to 2400 × 10^6^, while ERα mRNA ranged from 1.0 to 620,000 × 10^6^. The ratios of ERβ to ERα mRNA in BCa ranged from 1:1 to 1:300. ERα mRNA was detected in 93.54% of BCa, whereas ERβ1, 2, and 5 isoform mRNA was detected in 60.9%, 52.9%, and 41.3% of the cohort, respectively. ERβ1 mRNA significantly correlated with ERα mRNA expression (*r* = 0.27, *p* = 0.002) and ERα protein expression (*r* = 0.18, *p* = 0.038), and it was co-expressed with ERα in 67% of BCa. All three (ERβ1, ERβ2, and ERβ5) isoforms were detected in 14.5% (20/138) of tumors, while no ERβ isoforms were detected in 18.8% (26/138). Two or three ERβ isoforms were co-expressed in 68.8% (95/138) of BCa. The ERβ1 isoform was more frequently expressed cohort-wide (*p* = 0.0007), while ERβ2 expression was more frequent than that of ERβ5 in HER2/neu-positive tumors (*p* = 0.007), and HER2 molecular type (*p* = 0.007) in McNemar’s test. 

**Figure 1:**
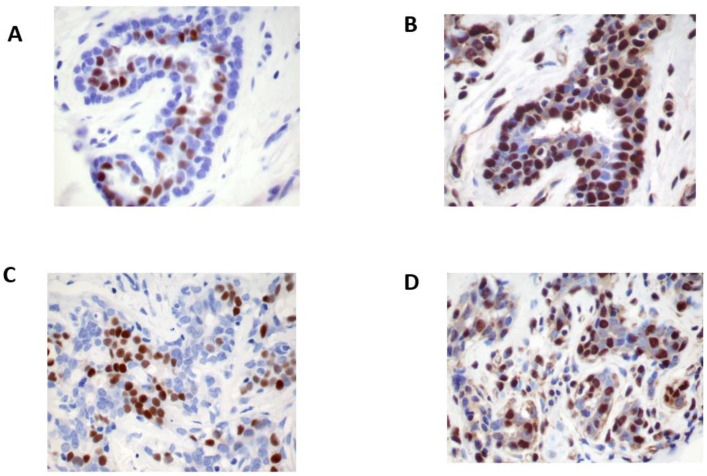
Immunohistochemistry stains of estrogen receptor (ER) β protein expression in normal and benign breast tissue. ERβ expression (**A**) is expressed in the nuclei of benign epithelial cells and myoepithelial cells, stromal cells, and lymphocytes, whereas ERα (**B**) is expressed only in the nuclei of epithelial cells. The ERβ reaction (**C**) is abundant and stronger than that of ERα (**D**) (immunohistochemistry stain using polyclonal ERβ1 (385p/AR385-10R) antibody, original magnification ×20).

**Figure 2:**
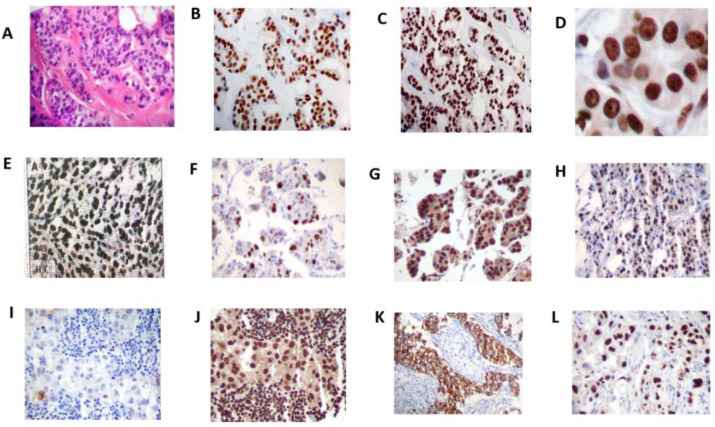
H&E stains of infiltrating duct carcinoma (**A**); immunohistochemistry stains of ERα (**B**) showing positive nuclear reaction only in neoplastic epithelial cells; ERβ expression (**C**) exhibiting strong and diffuse immunoreaction of the nuclei of neoplastic epithelial cells and stromal cells (original magnification ×20) (**D**); diffuse and intense staining of ERβ expression in the nuclei (original magnification ×40); differential expression of ERβ in breast cancer types; ILC (**E**), infiltrating lobular carcinoma; APO (**F**), apocrine carcinoma; MIC (**G**), micropapillary carcinoma; MUC (**H**), mucinous carcinoma. ERα-negative BCa (**I**) showing high ERβ expression (**J)**; co-expression of ERβ with high Her-2/neu positivity (**K**) and high P53 expression (**L**). Original magnification ×20.

In the ERβ IHC study, the ERβ isoform protein was strongly positive in the nuclei of luminal epithelial and myoepithelial cells, fibroblasts, endothelial cells, and lymphocytes in benign breast tissues, whereas ERα protein was positive only in the nuclei of epithelial cells (Figure 1). ERβ1 antibodies of clones 14C8, PA1-313, and PPG5/10 showed inconsistent immune reactivity. PPG5/10 antibodies from two different vendors displayed discordant reactions, and 14C8 presented lower levels of detection than other ERβ1 antibodies. The polyclonal ERβ1 (385p/AR385-10R) and ERβ5 (57/3) antibodies produced strong nuclear staining but also some cytoplasmic staining. The polyclonal ERβ1 (385p/AR385-10R) antibody exhibited the most consistent reaction after a careful titration of the antibody up to 1:800 dilution and overnight incubation. Thus, all ERβ1 protein expression studies in BCa and BCC were conducted using the polyclonal (385p/AR385-10R) ERβ1 antibody (Figure 1 and Figure 2). Under the optimum immunostaining conditions, the immunoreaction by ERβ2 and 5 antibodies (Table 1) also displayed the same intensity of nuclear staining as that of ERβ1 protein expression. ERβ isoform 1, 2, or 5 protein expression was detected in 61.5%, 44.9%, and 59.5% of the cohort, respectively. ERβ1 protein expression (Figure 2) using 385p/AR385-10R ERβ antibody showed differential expression in BCa subtypes, with a higher expression in well-differentiated duct BCa and lobular carcinoma than poorly differentiated BCa, and a high ERβ1 protein expression in ERα-negative BCa with high co-expression of HER2/neu and p53 (Figure 2I–L).

When assessing the relationship between immunostaining and qRT-PCR for paired samples from each case, overall, the majority of cases with high ERβ mRNA levels also had high levels of ERβ protein. The level and rate of ERα protein expression were highly correlated with ERα mRNA (*r* = 0.41, *p* < 0.0001) and those of ERβ5 protein expression were highly correlated with ERβ5 mRNA in the entire cohort (*r* = 0.27, *p* = 0.0015), ERα-positive BCa (*r* = 0.32, *p* = 0.006), TNBC (*r* = 0.9, *p* = 0.051) and ERα-negative BCa (*r* = 0.21, *p* = 0.09). However, ERβ1 (*r* = 0.004, *p* = 0.96) and ERβ2 (*r* = 0.05, *p* = 0.605) protein expression was not correlated with their mRNA expression in the entire cohort of BCa. Overall, the levels and rates of ERβ isoform protein and mRNA expression were consistent with 39.1% for ERβ1, 40.6% for ERβ2, and 53% for ERβ5. 

Hence, there was a subset of cases with concomitant high ERβ mRNA and protein expression and another subset of cases in which high protein levels were not accompanied by high mRNA levels. When comparing ERβ protein expression with that of ERα, ERβ1 protein expression correlated with ERα protein expression (*r* = 0.18, *p* < 0.039), but ERβ2 and ERβ5 expression did not. However, each ERβ protein expression was significantly correlated with another ERβ isoform (*r* = 0.34, *p* < 0.0001). 

### 3.2. ERβ Isoform mRNA and Protein Expression Is Differentially Associated with Clinical Characteristics and Molecular Types in BCa

ERβ mRNA and protein expression was differentially associated with clinical characteristics and BCa subtypes according to Fisher’s exact test (Table 2 and Table 3). High ERβ1 protein expression was significantly associated with large tumors (>2 cm), while ERβ2 protein expression was significantly associated with node-positive tumors (Table 2). High ERβ2 mRNA expression was inversely correlated with luminal A type BCa. High ERβ5 protein was correlated with basal-like and HER2 type BCa (Table 3). However, there was no significant association between ERβ isoform mRNA or protein expression and grade, PR, HER2/neu, Ki-67, or p53 expression.

### 3.3. ERβ Isoform mRNA and Protein Expression Is Associated with High Ki-67 Positivity

As shown in the Spearman rank correlation test (Table 4), ERβ isoform mRNA or protein expression in various BCa subtypes of BCa including molecular types and subgroups was associated with >15% Ki-67 positivity. High ERβ1 and ERβ2 protein expression in ERα-negative BCa, and ERβ1 and ERβ5 protein expression in TNBC and ERα-positive BCa were associated with >15% of Ki-67 positivity. The following cases were also associated with >15% Ki-67 positivity: ERβ1 protein expression in luminal A type, basal-like type, and high-grade tumors; ERβ5 protein expression in luminal B type, HER2 type, high-grade, large-size, and P53-positive tumors; ERβ2 protein and luminal A type BCa; ERβ2 mRNA and ERβ5 mRNA expression in HER2 type BCa; ERβ5 mRNA expression in high-grade and HER2/neu-positive BCa. The findings suggest that ERβ-expressing BCa cells are proliferating cells.

### 3.4. ERβ Isoform Protein and mRNA Expression and Overall Survival in the BCa Subtypes and Subgroups

Overall, the association between ERβmRNA and protein expression and OS was more notable in the patient subgroups than in the entire cohort. The mRNA or protein expression of each ERβ isoform appeared to be distinctly associated with OS and clinical characteristics.

**Figure 3:**
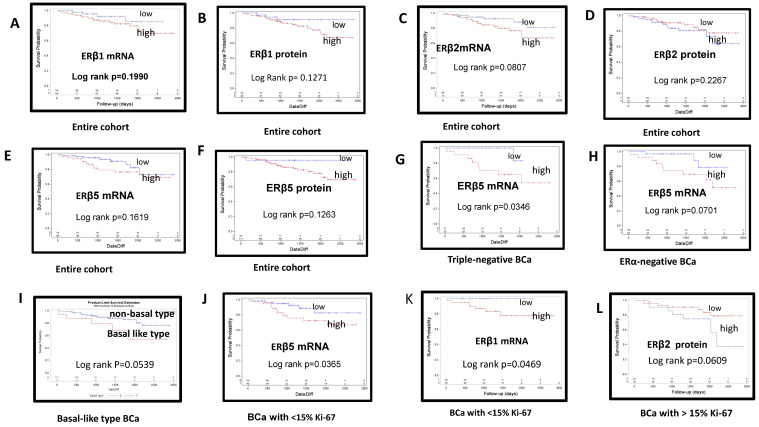
Kaplan–Meier curves of overall survival estimates stratified by estrogen receptor (ER) β mRNA and protein expression in breast cancers: (**A**) high vs. low ERβ1 mRNA in the entire cohort; (**B**) high vs. low ERβ1 protein in the entire cohort; (**C**) high vs. low ERβ2 mRNA in the entire cohort; (**D**) high vs. low ERβ2 protein in the entire cohort; (**E**) high vs. low ERβ5 protein in the entire cohort; (**F**) high vs. low ERβ5 protein in the entire cohort; (**G**) high vs. low ERβ5 mRNA expression in patients with triple-negative breast cancer; (**H**) high vs. low ERβ5 mRNA expression in ERα-negative BCa; (**I**) basal-like type BCa with trend of poor OS; (**J**) high or low ERβ5 mRNA expression in BCa with <15% Ki-67 staining; (**K**) high vs. low ERβ1 mRNA expression in BCa exhibiting <15% Ki-67 staining; (**L**) high or low ERβ2 protein expression in BCa exhibiting >15% Ki-67.

In KM log rank OS analysis (Figure 3), ERβ isoform 1, 2, or 5 mRNA and protein expression was associated with OS in the subgroups, but not in the entire cohort. High ERβ5 mRNA expression in ERα-negative BCa and TNBC was associated with poorer OS. High ERβ1 and ERβ5 mRNA expression in tumors with <15% Ki-67 positivity and high ERβ2 protein expression in tumors with >15% Ki-67 positivity were also predictive of poor OS. Basal-like type BCa showed a trend of poor OS. 

In the univariate cox PH analyses (Table 5), ERβ mRNA expression was associated with poor patient outcomes in the subtypes and subgroups. ERβ2 mRNA expression in ERα-negative BCa, HER2/neu-negative and PR-negative, high-grade, large-size (>2 cm), <15% Ki-67-positive and >5% p53 expression, and node-negative tumors was associated with poor survival outcomes. ERβ5 mRNA in TNBC and node-negative BCa was also associated with a risk of poor OS. In the entire cohort, high ERβ2 mRNA expression, large-size (>2 cm) tumors, and tumors with high P53 (>5%) positivity were risk factors for poor outcome. In contrast, ERβ2 protein expression in high-grade tumors and node-negative BCa was associated with favorable OS.

In Cox regression multivariate analysis with other clinicopathological parameters (Table 6), there was a trend of poor survival in tumors expressing ERβ5 mRNA (*p* = 0.063, HR 1.015, 95% CI 0.99–1.031) and a trend of better survival outcome in tumors expressing ERβ2 protein (*p* = 0.061, HR 0.98, 95% CI 0.958–1.001).

### 3.5. ERβ 2 and 5 Isoform Expression Is Predictive of Poor OS in ERα-Negative BCa and TNBC

ERβ1, 2, and 5 isoform mRNA was detected in 67.7%, 53.8%, and 43.0% of ERα-negative BCa and 58.1%, 60.0%, and 53.4% of TNBC, respectively. ERβ protein expression was lower than that of mRNA but was also highly and more frequently expressed for ERβ2 compared to ERβ5 (*p* = 0.0357) according to McNemar’s test. The association between high ERβ1 and ERβ2 protein expression and ERα-negative BCa, and between ERβ1 and ERβ5 protein expression and TNBC with >15% Ki-67 positivity (Table 4) suggests a potential role of ERβ in tumor growth in ERα-negative BCa and TNBC. High ERβ1 protein-expressing epithelial cells in ERα-negative BCa highly co-expressed HER2/neu and p53 (Figure 2I–L). In KM analysis (Figure 3), high ERβ5 mRNA expression in TNBC and ERα-negative BCa was predictive of poorer OS. In univariate Cox OS PH analysis (Table 5), high ERβ2 and ERβ5 mRNA expression in ERα-negative BCa, and high ERβ5 mRNA expression in TNBC were also predictive of poorer OS.

### 3.6. ERα and ERβ Isoform mRNA and Protein Expression in BCC Mirrored That in BCa

BCCs derived from different types of BCa served as an excellent control system for mRNA and protein analysis of different types of BCa. ERβ isoform mRNA or protein expression was high in luminal A and B type-derived BCC, but low in basal-like type BCCs. ERβ and ERα mRNA and protein were co-expressed in BCCs derived from luminal type BCa. Fresh and formalin-fixed BCCs yielded comparable levels of ERβ mRNA expression.

## 4. Discussion

While the tumor-promoting actions of ERα are well known, ERβ has been shown to act as an oncosuppressor. The exact role of ERβ in carcinogenesis and tumor progression is not yet fully understood. Highly variable and even opposite effects have been ascribed to ERβ in BCa, including both proliferative and growth-inhibitory actions. Overall, the outcome results of the studies are inconsistent. The mRNA and protein expression of ERβ isoforms in BCa is associated with favorable or adverse clinical outcomes and beneficial or poor responses to endocrine therapy [12,13,14,15,16]. The inconsistent and controversial results may be due to the complexity of ERβ isoforms as a function of their post-translational modification, study subject heterogeneity, and varied study designs targeting ERβ mRNA or protein expression or different ERβ isoforms. However, they might also be due to the lack of a standardized testing protocol for ERβ mRNA or protein expression.

### 4.1. ERβ1, 2, and 5 Isoforms Are Differentially Associated with Clinical Outcomes in BCa

Many studies (Table 7) have shown that each ERβ isoform is differentially associated with favorable or adverse clinical outcomes. The observed favorable outcomes included increased OS, disease-free survival (DFS), association of good prognostic markers, and beneficial TAM responses in patients whose tumors expressing ERβ1 [31,32,33,34,35,36,37,38,39,40], ERβ2 [31,41,42,43,44,45,46], and ERβ5 [41,45]. High ERβ2 mRNA was associated with a favorable TAM response [43]. Higher ERβ1 protein expression was detected in luminal A and B type BCa than HER2 or basal-like types [35]. Adverse outcomes were observed in tumors expressing high ERβ1 [36,47,48,49,50,51,52,53,54,55,56], ERβ2 [41,57,58,59,60,61,62,63,64], and ERβ5 [60,65]. High ERβ2 cytoplasmic expression without nuclear expression was associated with worse outcome [41,57]. Early disease recurrence and poor response to TAM have been observed in tumors with high ERβ2 protein expression but low PR expression in a neoadjuvant setting [59]. High ERβ exon 5 splice variant mRNA was detected in grade III tumors in postmenopausal women [64]. In ERα-negative BCa and TNBC, ERβ isoforms are highly detectable, associated with high Ki-67 positivity; they have also been implicated in the growth of BCa, independent of estrogen or growth factors. Clinical studies on the expression of different ERβ isoforms in ERα-negative BCa and TNBC also showed favorable or adverse outcomes. Favorable outcomes were observed in patients with tumors expressing ERβ1 [57,66,67,68]. Adverse outcomes were observed in patients with tumors expressing ERβ1 [47,69,70,71,72,73,74,75,76], ERβ2 [57,69,77], and ERβ5 [76,78]. ERβ2 cytoplasmic expression in basal-like BCa was associated with shorter survival in familial BCa [57]. A high level of ERβ5 mRNA in patients with ERα-negative BCa in African American women was considered to contribute to poor survival, and this might be related to the estrogen-independent transcriptional properties of the ERβ5 isoform [7]. Similar to the observations in BCa, high ERβ5 protein expression in prostate carcinoma was shown to be associated with poor survival and invasiveness [79].

### 4.2. ERβ mRNA or Protein Expression Is Differentially Associated with Clinical Outcomes in BCa

ERβ isoform protein or mRNA expression in BCa was shown to be associated with either favorable or adverse outcomes. Many studies (approximately 60–70% of studies) reported that ERβ isoform protein expression is associated with favorable outcomes including increased OS and DFS, as well as positive responses to endocrine therapy. However, some studies reported that ERβ isoform protein or mRNA expression is associated with poor outcomes or no association with outcomes [12,13,14,15,16]. Tan et al. observed differential outcomes in BCa types; in patients with ERα-positive tumors, ERβ protein positivity was not associated with DFS or OS but it was associated with increased DFS or OS in ERα-negative patients, while there was no association between ERβ mRNA levels and DFS and OS.

The studies on ERβ isoform mRNA expression in BCa are limited. The studies reviewed herein observed favorable outcomes in some studies [31,43,45,46], but adverse clinical outcomes including poor prognostic markers and poor response to TAM in many more studies [7,47,48,52,53,56,60,63,64,75,76,80]. ERβ2 and ERβ5 mRNA expression was associated with significantly better relapse-free survival (RFS), while ERβ1 mRNA expression was not associated with any measure of OS [45]. Patients with high expression of ERβ1 mRNA or ERβ2 mRNA had a significantly better DFS and OS than those with low expression [31]. ERβ2 mRNA levels were significantly associated with better outcome in ERα-positive BCa and in node-negative tumors, while high ERβ mRNA and protein expression was associated with a significantly better outcome [43]. Higher levels of ERβ2 than ERβ1 isoform were associated with a better outcome in late-onset patients [46].

In contrast, as adverse outcomes, ERβ2/βcx mRNA levels were increased during growth and progression of BCa [63]. A high level of ERβ5 mRNA in African American patients with ERα-negative BCa was considered to contribute to their poor survival [76]. ERβ1 positivity (according to RT-PCR) in ERα-negative BCa led to larger tumors and higher-stage BCa than ERβ1 positivity in ERα-positive BCa [47]. Positive ERβ mRNA predicted higher recurrence and death rates [48] and high-grade tumors [56]. ERβ mRNA was significantly upregulated in the TAM-resistant group as compared with the tamoxifen-sensitive group [52]. ERβ mRNA was higher in tumors in the TAM-resistant group and highly Ki-67-positive tumors than in those from the control group [53]. Higher ERβ2 mRNA than ERβ1 or ERβ5 mRNA expression was correlated with the level of tumor inflammation and tumor grade [60]. ERβ exon 5△ mRNA levels were significantly increased in grade III tumors and in tumors of postmenopausal women [64]. An absolute and relative increase in ERβ mRNA levels in ERα-negative and PR-negative BCa suggested a possible involvement of upregulation of ERβ mRNA in the development of estrogen-independent tumors [75]. Kim et al. [80] demonstrated that ERβ mRNA expression according to branched-chain QuantiGene2.0 assay using FFPE was associated with worse DFS, as well as poorly differentiated, lymph node-positive, and PR-negative tumors; ERβ mRNA is, thus, considered an independent predictor of disease recurrence in ERα-positive BCa.

When ERβ protein and mRNA expression in BCa was investigated simultaneously, the levels of ERβ mRNA were not consistent with ERβ protein expression whether the studies were conducted using frozen breast cancer tissue [31,47,81] or archived FFPE breast cancer tissue [43,80]. The levels of ERβ mRNA expression correlated with ERβ protein levels in 34–54% of the cases [43,47], similar to our study. The clinical outcomes of ERβ mRNA or protein expression were not always consistent [43,47,63,80,81]. ERβ mRNA was associated with worse DFS, as well as poorly differentiated, lymph node-positive, and PR-negative tumors, in ERα-positive BCa, whereas ERβ1 protein was associated with smaller tumors [80]. High ERβ2 mRNA was associated with a favorable TAM response and improved survival in node-negative BCa, ERα-positive BCa, and the entire cohort of TAM -treated patients, whereas ERβ2 protein levels were associated with better outcome only in ERα-positive BCa [43]. Oneille et al. [47] demonstrated that ERβ1 protein and mRNA levels were inconsistent (*p* = 0.08). ERβ1 mRNA (according to RT-PCR) showed no association with outcome, while ERβ1 protein expression showed a trend for a worse outcome in all cases, as well as in ERα-positive tamoxifen-treated cases. High ERβ total protein expression was associated with TAM-sensitive tumors, whereas ERβ 1, 2, and/or 5 mRNA expression was not [81]. Such discrepant findings were reported by the same research group in two different studies; in the first study, they reported TAM resistance in tumors expressing high ERβ mRNA [52], while, in the second study, they demonstrated better DFS in patients on exemestane therapy with low ERβ1 protein expression, as well as better DFS in patients on TAM therapy in tumors with high ERβ1 protein expression [39]. High mRNA and low protein levels may have been due to the fact that mRNA was analyzed in tissue homogenates containing other cell types, whereas IHC immunostaining results were evaluated only on epithelial cells. Furthermore, the different clinical outcomes observed in studies on ERβ mRNA or protein expression may partly have been due to varied testing protocols for ERβ mRNA expression (RT-PCR) or protein expression (IHC).

### 4.3. ERβ as a Potential Therapeutic Target in BCa

ERβ expression has been shown to be associated with favorable or adverse clinical outcomes; hence, agonists or antagonists to ERβ or downstream targets have been suggested as potential therapeutic targets [17,18,19]. ERβ expression has been associated with good or poor responses to endocrine therapies. High ERβ1 protein expression in patients with ERα-positive or ERα-negative BCa or TNBC tumors was predictive of a good response to TAM therapy [31,34,39,57,66,68]. ERβ2 mRNA expression was associated with a favorable TAM response and with significantly improved relapse-free survival (RFS) and OS [43], while ERβ5 mRNA expression was associated with improved RFS in a subset of patients receiving TAM [45]. High ERβ1 nuclear expression in tumors in familial BCa was predictive of TAM therapy response [57], and it was a significant discriminating factor for DFS in node-negative luminal A type BCa, predicting the response to hormonal therapy [36]. High ERβ2 protein expression is associated with a favorable response [31]. High Ki-67 positivity (>15%) in ERβ-expressing cells with a high proliferation rate might render the cells more sensitive to TAM. In contrast, ERβ protein expression was indicative of a poor response or resistance to TAM therapy in patients with tumors expressing high levels of ERβ1 protein [47,55,74], tumors expressing high levels of ERβ2 protein [58], and tumors expressing high levels of ERβ2 protein with low levels of PR in a neoadjuvant setting [59]. ERβ mRNA expression was significantly upregulated in the TAM-resistant group as compared with the TAM-sensitive group [52]. ERβ1 protein expression was associated with a trend of worse RFS outcome in all cases, as well as in ERα-positive TAM-treated cases [47]. Furthermore, ERβ2/ERβ5 and ERβ1 have exhibited sharply contrasting activities in TNBC cells. ERβ2 and 5 exhibited pro-oncogenic activities in TNBC; thus, the development and clinical use of specific antagonists can be applied in TNBC treatment, while ERβ1 activation might be used to limit the growth and spread, as well as to increase the drug sensitivity, of TNBC [82]. This implies that delineating the absolute amounts and relative ratios of the different ERβ isoforms might have prognostic and therapeutic relevance, and it could enable better selection of optimal approaches for treatment of this often aggressive form of BCa.

### 4.4. ERβ Studies in Breast Cancer Cell Lines (BCCs)

Studies on ERβ in BCCs derived from ERα-negative BCa or TNBC have also shown contrasting growth-inhibitory or -stimulatory effects [82,83,84,85,86].

### 4.5. Variation in ERβ mRNA and ERβ Protein Testing Protocols

The studies reviewed herein showed variable ERβ protein or mRNA testing protocols. The validation methods of IHC involving primary and secondary antibodies, visualization systems, equipment, and controls were not consistent. Immunohistochemistry studies were conducted using a wide range of commercially available monoclonal and polyclonal ERβ antibodies or in-house developed antibodies with or without in vivo validation. ERβ2 isoform protein expression has been analyzed using clone 57/3 and other polyclonal ERβ2 antibodies. ERβ5 isoform protein expression has been investigated using clone 5/25 and other ERβ5 antibodies. ERβ1 isoform protein expression has been analyzed using different clones of ERβ1 antibodies, both single and combined, such as PPG5/10, 14C8, PA313, polyclonal ERβ1 (385p/AR385-10R), and in-house-raised antibodies. The 14C8, PA1-313, and PPG5/10 ERβ1 antibodies reportedly yield high and specific detection levels of full-length ERβ, but they seemed to only produce reliable results in some studies [23,24]. Wu et al. [24] reported that the subcellular localization of ERβ as detected by the PPG5/10 and MC10 antibodies is variable. Increased levels of cytoplasmic staining as detected by the MC10 antibody are likely explained by the presence of ERβ variants 3–5, while the significance of cytoplasmic localization of ERβ antibodies may hinder an assessment of their sensitivity and specificity in the absence of thorough characterization. The differences in nuclear staining between PPG5/10 and MC10 antibodies was explained by the fact that the PPG5/10 antibody recognizes the C-terminal end, while the MC10 antibody recognizes the N-terminal region. Further research is needed to determine whether such staining patterns in BCa could be of predictive and/or prognostic value. The cutoff threshold to define ERβ staining positivity for ERβ protein expression varies significantly, with the detection rate of ERβ positivity ranging from 15.9% to 92.0%. Thus, the results of many studies on ERβ protein expression have varied [87].

Studies on ERβ mRNA studies have been conducted using fresh tumor tissues [47,48,52,53,54,63,76], as well as archived formalin-fixed tissues [43], by RT-PCR or by branched-chain QuantiGene2.0 assay [80,88]. Total ERβ/ERβ1 was most frequently analyzed, and more than one ERβ isoform mRNA was analyzed including ERβ2, ERβΔ5, and ERβ5 mRNA. Although ERβ mRNA measurement can provide a more accurate determination of ERβ at the molecular level, ERβ mRNA analysis has drawbacks for routine application. The mRNA expression may not necessarily reflect protein expression, and it can be degraded to undetectable levels during processing or become contaminated with stromal cells on disaggregated tissue preparations. ERβ mRNA from other cell types might account for a positive RT-PCR but negative IHC. 

## 5. Conclusions

Our study was a comprehensive, simultaneous investigation of the mRNA and protein expression of ERβ1, ERβ2, and ERβ5 isoforms, in the entire cohort, as well as in various subtypes and subgroups of BCa. The main findings of our studies were that ERβ isoforms and their mRNA and protein expression differ and are associated with different clinical outcomes in subtypes and subgroups. ERβ2 and ERβ5 mRNA expression is predictive of poor outcomes in ERα-negative BCa and TNBC. Overall, ERβ isoform-expressing BCa cells are proliferating cells exhibiting high Ki-67 positivity. The findings in our study are consistent with some previous studies demonstrating adverse outcomes associated with high ERβ expression [41,48,54,55,58,61,65,69,72,74,77,80,82]. Our study was limited by its relatively small sample size in some subgroups, as well as a lack of endocrine therapy responses and the usage of commercial ERβ antibodies without in vivo validation for ERβ protein assays.

Our study and previous studies reviewed herein demonstrated that the mRNA or protein expression of different ERβ isoforms seemingly plays a significant role in favorable or adverse outcomes in BCa. The inconsistent clinical outcomes observed may be related to many factors, while they may also be partly related to the lack of a standardized testing protocol. Thus, standardizing ERβ mRNA or protein testing and evaluation protocols by setting a cutoff value of ERβ positivity may be needed for consistent and reproducible measurements of ERβ expression to investigate its clinical relevance in BCa, as seen for ERα [26,89,90]. Standardizing ERβ testing would facilitate its clinical application in BCa [17,18,19]. Further investigation of ERβ isoforms in a large cohort of BCa subgroups is warranted to explore the role of ERβ as a prognostic and predictive factor, and as a potential therapeutic target in BCa.

## Figures and Tables

**Table 1:** List of antibodies used for immunohistochemistry.

Antibody	Antibody Clone	Supplier
**ERβ1**	Ab288/14C8	Abcam Inc, Cambridge, UK
**ERβ1**	385P/AR 385-10R	Biogenex, San Ramon, CA, USA
**ERβ1**	MCA1974S/PPG5/10	DAKO, Carpintena, CA, USA
**ERβ1**	PAI-313	ThermoFisher Scientific, Rockford, IL, USA
**ERβ2**	MCA2279S/57/3	Bio-Rads, Hercules, CA, USA
**ERβ5**	MCA4676/5/25	Bio-Rads, Hercules, CA, USA
**CK5/6**	D5/6	DAKO, Carpintena, CA, USA
**Ki-67**	MIB-1	DAKO, Carpintena, CA, USA
**P53**	D07	DAKO, Carpintena, CA, USA
**EGFR**	3C6	DAKO, Carpintena, CA, USA
**HER2/neu**	HerceptTest	DAKO, Carpintena, CA, USA
**ERα**	ID5	DAKO, Carpintena, CA, USA
**Vimentin**	V9	DAKO, Carpintena, CA, USA
**Cytokeratin**	AE1/AE3	DAKO, Carpintena, CA, USA
**PR**	Pg363	DAKO, Carpintena, CA, USA

**Table 2:** Associations between ERβ isoform mRNA and protein expression and clinical characteristics.

		ERβ1 mRNA			ERβ1 Protein			ERβ2 mRNA			ERβ2 Protein			ERβ5 mRNA			ERβ5 Protein		
Variables		Pos	Neg	*p*-Value	Pos	Neg	*p*-Value	Pos	Neg	*p*-Value	Pos	neg	*p*-Value	Pos	Neg	*p*-Value	Pos	Neg	*p*-Value
**ERα status**	Pos	38	34	0.0548	50	22	0.477	34	38	0.176	44	27	0.39	29	43	0.863	57	15	0.508
	neg	46	20		42	24		39	27		35	31		28	38		56	10	
**Her-2/neu**	Pos	27	16	0.851	31	12	0.437	27	16	0.142	21	22	0.198	13	30	0.0954	34	9	0.635
	Neg	57	38		61	34		46	49		58	37		44	51		79	10	
**PR**	Pos	34	30	0.159	45	22	1	30	36	0.125	39	28	0.864	27	39	1	52	15	0.269
	Neg	47	24		47	24		42	29		40	31		29	42		61	10	
**Ki-67**	>15%	19	10	0.67	23	6	0.123	14	15	0.779	20	9	0.205	16	13	0.095	26	3	0.285
	<15%	65	44		68	40		59	50		59	49		41	68		87	22	
**Grade**	Grade 2/3	74	46	0.6156	83	37	0.178	66	54	0.217	69	51	1	52	68	0.305	99	20	0.323
	Grade 1	10	8		9	9		7	11		10	8		5	13		13	5	
**Tumor size**	>2 cm	31	23	0.592	42	12	**0.0282**	30	24	0.727	32	22	0.727	23	31	0.86	47	7	0.26
	<2 cm	53	31		50	34		43	41		47	37		34	50		66	18	
**Nodal status**	Pos	17	14	0.531	24	7	0.195	21	10	0.0685	23	8	**0.0392**	11	20	0.536	29	2	0.0654
	Neg	67	40		68	39		52	55		56	51		46	61		84	23	
**CK5/6**	Pos	9	7	0.787	10	6	0.779	9	7	0.797	9	7	1	8	8	0.59	16	0	0.0764
	Neg	75	47		82	40		64	58		70	52		49	73		97	25	
**P53**	Pos	26	28	0.163	27	19	0.717	40	25	0.231	37	22	0.17	45	36	1	17	8	0.19
	Neg	51	33		50	42		37	36		40	39		32	25		60	53	

Bold: significant *p*-value < 0.05.

**Table 3:** Associations between ERβ isoform mRNA and protein expression and molecular types.

	ERβ1 mRNA			ERβ1 Protein			ERβ2 mRNA			Erβ2 Protein			ERβ5 mRNA			ERβ5 Protein		
Types	Pos	Neg	*p*-Value	Pos	Neg	*p*-Value	Pos	Neg	*p*-Value	Pos	Neg	*p*-Value	Pos	Neg	*p*-Value	Pos	neg	*p*-Value
**Luminal A type (50)**	27	23	0.276	33	17	1	20	30	**0.0328**	34	16	0.0732	21	29	1	40	10	0.653
	57	31		59	29		53	35		45	43		36	52		73	15	
**Luminal B type (25)**	14	12	0.505	19	7	0.497	17	9	0.193	12	14	0.271	9	17	0.511	18	8	0.0875
	70	42		73	39		56	56		67	45		46	64		95	17	
**Basal-like type (17)**	10	7	1	11	6	1	10	7	0.796	11	6	0.605	9	8	0.307	17	0	**0.0419**
	74	47		81	40		63	58		68	53		48	73		96	25	
**HER2 type (17)**	12	5	0.109	11	6	1	8	9	0.616	8	0	0.436	7	10	1	17	0	**0.0418**
	68	44		81	40		71	50		71	50		49	71		95	25	
**TNBC (43)**	31	13	0.137	29	17	0.439	26	18	0.363	24	20	0.713	23	21	0.0951	39	5	0.235
	53	41		65	27		47	47		55	39		34	60		74	20	

Bold: significant *p*-value < 0.05.

**Table 4:** Spearman rank-order correlation between ERβ isoform mRNA and protein expression and KI -67 > 15% positivity in breast cancer subgroups.

	ERβ1 mRNA	ERβ1 Protein	ERβ2 mRNA	ERβ2 Protein	ERβ5 mRNA	ERβ5 Protein
**Breast Cancer Subgroups (Cases)**	rho (*p*-Value)	rho (*p*-Value)	rho (*p*-Value)	rho (*p*-Value)	rho (*p*-Value)	rho (*p*-Value)
**ERα + and ERα- (138)**	0.15 (0.86)	**0.38 (<0.0001)**	0.096 (0.26)	0.14 (0.088)	**0.18 (0.032)**	**0.34 (0.0001)**
**ERα + (73)**	−0.077 (0.52)	**0.28 (0.017)**	0.011 (0.93)	0.032 (0.79)	0.16 (0.21)	**0.272 (0.021)**
**ERα- ( 65 )**	0.107 (0.39)	**0.42 (0.005)**	0.17 (0.18)	**0.31 (0.012)**	0.22 (0.073)	**0.25 (0.041)**
**TNBC (43)**	0.03 (0.85)	**0.45 (0.0021)**	0.09 (0.55)	0.19 (0.23)	0.104 (0.50)	**0.31 (0.039)**
**Luminal A type (50)**	−0.13 (0.38)	**0.39 (0.0052)**	**−0.23 (0.0084)**	**0.19 (0.027)**	0.22 (0.13)	0.21 (0.13)
**Luminal B type (25)**	−0.1 (0.65)	0.11 (0.59)	−0.26 (0.19)	0.10 (0.62)	0.06 (0.75)	**0.30 (0.014)**
**HER2 type (17)**	0.07 (0.68)	0.36 (0.16)	**0.52 (0.0060)**	0.250 (0.010)	**0.49 (0.045)**	**0.48 (0.048)**
**Basal-like type (17)**	−0.084 (0.75)	**0.62 (0.0081)**	−0.016 (0.99)	0.39 (0.14)	0.178 (0.49)	0.12 (0.65)
**Grade 2/3 tumors (125)**	**0.28 (0.023)**	**0.38 (<0.0001)**	0.009 (0.92)	0.17 (0.071)	**0.189 (0.041)**	**0.33 (0.0003)**
**>2 cm tumor (40)**	0.102 (0.48)	**0.32 (0.017)**	0.07 (0.61)	0.20 (0.15466)	0.07 (0.61)	**0.33 (0.014)**
**Her2/neu+ (39)**	0.06 (0.68)	0.255 (0.89)	−0.14 (0.35)	0.25 (0.10)	**0.33 (0.031)**	**0.31 (0.045)**
**p53>5%**	0.07 (0.59)	**0.36 (0.0034)**	−0.03 (0.81)	−0.01 (0.93)	0.12 (0.3481)	**0.30 (0.0175)**

r (rho): rank, Bold: significant *p*-value < 0.05, TNBC: triple negative BCa.

**Table 5:** Cox univariate analysis of ERβ Isoform expression and overall survival in breast cancer subtypes and subgroups and in the entire cohort.

	ERβ1 mRNA		ERβ1 Protein		ERβ2mRNA		ERβ2 Protein		ERβ5 mRNA		ERβ5 Protein	
Subgroups (case#)	*p*-Value	HR( CI)	*p*-Value	HR(CI)	*p*-Value	HR(CI)		HR(CI)	*p*-Value	HR(CI)	*p*-Value	HR(CI)
**ERα-positive BCa(73)**	0.88	1.12 (0.27–4.53)	0.98	1.02 (0.25–4.06)	0.24	2.22 (0.59–8.3)	0.106	0.32 (0.08–1.23)	0.93	1.07 (0.28–4.10)	0.75	0.997 (0.98–1.02)
**ERα-negative BCa(65)**	0.33	1.79 (0.56–5.72)	0.21	2.7 (0.59–12.21)	**0.034**	**3.59 (1.10–11.72)**	0.41	0.61 (0.19–1.95)	**0.09**	**3.22 (0.8512.2)**	**0.072**	**1.03 (0.997–1.06)**
**TNBC (43)**	0.12	3.03 (0.75–12.22)	0.36	2.1 (0.43–10.2)	0.056	4.005 (0.96–16.63)	0.25	0.98 (0.96–1.01)	**0.069**	**6.98 (0.83–56.45)**	0.26	1.02 (0.99–1.05)
**TNBC- (95)**	0.83	0.87 (0.23–2.95)	0.6	1.42 (0.38–5.26)	0.24	1.98 (0.63–6.23)	0.24	0.49 (0.16–1.58)	0.89	0.92 (0.28–3.03)	0.5	1.006 (0.98–1.02)
**Her2/neu+ (39)**	0.78	1.249 (0.28–5.59)	0.89	1.11 (0.21–5.82)	0.51	1.67 (0.37–7.530	0.29	0.419 (0.08–2.2)	0.88	1.13 (0.24–5.28)	0.73	0.98 (0.98–1.01)
**Her2/neu- (99)**	0.33	1.71 (0.59–4.950	0.3	1.96 (0.55–7.04)	**0.023**	**3.44 (1.198–9.93)**	0.16	0.47 (0.16–1.36)	0.13	2.53 (0.77–8.26)	0.077	1.02 (0.99–1.05)
**PR+ (54)**	0.58	0.63 (0.12–3.2)	0.67	1.42 (0.29–7.04)	0.58	1.59 (0.36–6.28)	o.24	0.42 (0.10–1.78)	0.74	1.28 (0.31–5.31)	0.58	1.006 (0.99–1.03)
**PR- (84)**	0.12	2.43 (0.78–7.48)	0.41	1.79 (0.47–6.35)	**0.01**	**4.59 (1.45–14.55)**	0.18	0.46 (0.15–1.43)	0.12	2.6 (0.78–8.6)	0.25	1.012 (0.99–1.03)
**Luminal A type (50)**	0.46	0.43 (0.05–4.03)	0.56	1.93 (0.22–17.4)	0.38	2.35 (0.37–13.8)	0.79	0.79 (0.13–4.73)	0.71	0.7 (0.11–4.47)	0.22	1.03 (0.98–1.07)
**Luminal B type (25)**	0.2	3.6 (0.51–25.59)	0.51	0.52 (0.07–3.69)	0.65	1.58 (022–11.23)	0.99	0.000 (0.00–1.5)	0.55	1.82 (0.26–12.93)	0.26	0.98 (0.96–1.01)
**HER2 type (17)**	0.63	0.99 (0.98–1.011)	0.209	1.03 (0.99–1.07)	0.967	0.99 (0.89–1.11)	0.078	1.061 (0.993–1)	0.71	0.94 (0.583–1.499)	0.34	1.213 (0.818–1.798)
**Basal type (17)**	0.54	1.77 (2.89–10.94)	0.35	1.012 (0.99–9.0)	0.15	4.29 (0.59–30.18)	0.65	0.66 (0.11–4.03)	0.26	3.53 (0.39–31.80)	0.91	0.99 (0.930–1.067)
**Grade 2/3 (115)**	0.55	1.31 (0.54–3.19)	0.6	1.31 (0.47–3.62)	**0.016**	**1.005 (1.001–1.010)**	0.17	0.99 (0.97–1.005)	0.29	1.63 (0.65–4.060	0.33	1.007 (0.99–1.02)
**Grade 1 (23)**	0.23	1.05 (0.97–1.13)	0.38	1.044 (0.95–1.2)	0.83	0.99 (0.962–1.032)	0.94	1.003 (0.93–1.08)	0.8	0.96 (0.71–1.31)	0.77	1.000 (0.96–1.06)
**>2 cm tumor (51)**	0.47	1.45 (0.53–3.95)	0.73	0.82 (0.26–2.57)	**0.035**	**2.88 (1.08–7.72)**	0.058	0.38 (0.14–1.04)	0.23	1.84 (0.68–6.04)	0.46	1.005 (0.97–1.007)
**<2 cm tumor (87)**	0.37	2.29 (0.37–14.06)	0.39	2.64 (0.29–23.8)	0.2	3.2 (0.53–19.20	0.5	0.54 (0.09–3.26)	0.18	4.62 (0.94–43.11)	0.45	1.01 (0.98–1.04)
**>15% Ki-67 (63)**	0.83	1.22 (0.19–7.44)	0.14	1.02 (0.99–1.06)	0.86	0.83 (0.09–7.45)	0.35	0.42 (0.065–2.66)	0.61	0.58 (0.07–4.77)	0.77	1.007 (0.96–1.56)
**<15% KI-67 (70)**	0.032	1.65 (0.61–4.42)	0.49	1.44 (0.50–4.16)	**0.014**	**3.56 (1.29–9.82)**	0.094	0.41 (014–1.17)	0.075	2.54 (0.91–7.07)	0.18	1.01 (0.99–1.027)
**LN positive (34)**	0.75	1.24 (0.33–4.65)	0.173	4.27 (0.53–34.3)	0.25	2.28 (0.55–9.43)	0.97	0.97 (0.24–3.95)	0.75	1.23 (0.33–4.65)	0.52	1.008 (0.98–1.034)
**LN negative (104)**	0.32	1.79 (0.57–5.63)	0.92	1.07 (0.32–3.57)	0.835	1.001 (0.993–1.008)	**0.019**	**0.16 (0.904–0.7)**	**0.024**	**5.86 (1.26–27.23)**	0.39	1.008 (0.99–1.03)
**p53>5% (57)**	0.28	2.01 (0.57–7.105)	0.973	1.000 (0.98–1.2)	**0.051**	**3.54 (0.99–12.56)**	0.066	0.29 (0.078–1.0)	0.61	0.99 (0.96–1.02)	0.33	1.017 (0.98–1.05)
**p53<5% (81)**	0.69	1.27 (0.38–4.19)	0.38	1.80 (0.48–6.8)	0.19	2.18 (0.66–7.16)	0.32	0.54 (0.16–1.85)	0.47	1.55 (0.46–5.19)	0.48	1.006 (0.98–1.023)
**ERα- + and ERα- BCa**	0.335	1.53 (0.64–3.65)	0.32	1.007 (0.993–1)	**0.022**	**2.72 (1.15–6.41)**	0.074	0.447 (0.19–1.0)	0.65	1.003 (0.992–1.0)	0.21	1.009 (0.99–1.02)

HR (CL); Hazard Ratio (Confidence Limit), Bold: signifcant *p*-value < 0.05.

**Table 6:** Multivariate cox PH analysis of ERβ expression and clinical characteristics for overall survival.

	*p*-Value	HR (95% CI)
**ERβ1 mRNA**	0.48	1.001 (0.998–1.006)
**ERβ2 mRNA**	0.12	1.006 (0.998–1.016)
**ERβ5 mRNA**	**0.063**	**1.015 (0.999–1.031)**
**ERβ1 protein**	0.65	1.005 (0.986–1.024)
**ERβ2 protein**	**0.061**	**0.98 (0.958–1.001)**
**ERβ5 protein**	0.097	1.019 (0.997–1.042)
**ERα**	0.73	1.005 (0.975–1.037)
TNBC *	0.67	0.424 (0.009–20.35)
Lum A type *	0.93	0.842 (0.016–42.99)
Lum B type *	0.75	1.672 (0.072–38.79)
HER2 type *	0.72	1.530 (0.152–15.38)
Basal Type *	0.53	0.209 (0.215–20.35)
HER2/neu *	0.94	1.158 (0.020–68.70)
Size of tumor **	**0.02**	**0.207 (0.055–0.778)**
Grade *	0.89	1.080 (0.338–3.451)
LN *	0.088	0.354 (0.107–1168)
**PR**	0.7	1.005 (0.982–1.028)
**Ki-67**	0.57	0.994 (0.972–1.016)
P53 **	**0.066**	**1.018 (0.999–1.037)**

Note: * positive; ** reference is >2 cm; CI, 95% confidence interval; variable without symbol, numerical.

**Table 7:** Association of ERβ isoform mRNA and protein expression with favorable and adverse clinical outcomes.

ERβ Isoform	High ERβ Expression with Favorable or Beneficial Outcome	# Cases	References	High ERβ Expression with Adverse or Poor Outcomes	# Cases	References
**ERβ1**	Increased DFS and OS, small size, low-grade and node-negative tumor	150	Sugiura [31]	Poor RFS, OS, and DFS in postmenopausal TAM-treated ERα^+^ BCa	138	O’ Neill [47]
	Increased DFS, RFS, and OS in Stage I and II BCa, with inverse correlation with HER2/neu	181	Nakopoulou [32]	Adverse survival outcome and recurrence with high ERβ mRNA	121	Markey [48]
	Increased DFS, inverse correlation with HER2/neu and SRC-1 expression	150	Meyers [33]	Worse prognosis and decreased tumor-free survival in endocrine therapy patients	589	Guo [49]
	Increased DFS in node-positive tumor	162	Zhang [34]	Recurrent BCa and node-positive BCa	120	Chang [50]
	Inverse correlation with HER2/neu^+^, CK56 and EGFR; no association with survival	2170	Marotti [35]	Reduced DFS, large tumor in postmenopausal endocrine-treated ERα^+^ BCa	195	Guo [51]
	Improved DFS in node-positive luminal A type	936	Novelli [36]	High-grade tumor, TAM resistance, LN-positive tumor	60	Speirs [52]
	Better TAM response	489	Iwase [37]	TAM resistance, and high Ki-67^+^ tumors with high ERβ1 mRNA expression	34	Chang [53]
	High ERβ1 was associated with low tumor size and 4 year DFS, while ERβ2 was associated with shorter DFS	1256	Speirs [39]	High EGFR positivity, TAM resistance	95	Knowlden [54]
	High ERβ1 was associated with better DFS	81	Dhimolea [38]	Upregulation of Ki -67 and cyclin A, recurrent BCa	29	Jenson [55]
	Association with favorable prognostic marker in chemotherapy-treated patients	1026	Elebro [40]	Poor DFS in luminal B type node-positive tumor	936	Novelli [36]
				High ERβ with worse DFS and poorly differentiated BCa	95	Kim [80]
**ERβ2**	Increased OS and DFS with nuclear ERβ2 expression	850	Shaaban [41]	Shorter survival	1256	Speirs [39]
	Increased DFS and OS with high ERβ2 mRNA	141	Sugiura [31]	Worse outcome in tumors with positive ERβ2 cytoplasmic expression/negative nuclear expression	757	Shaaban [41]
	Increased OS; low-grade tumor	150	Wurster [42]	Shorter or worse DFS and OS in TAM-treated patients	101	Baek [58]
	Increased DFS and OS in TAM-treated ERα^+^ BCa (ERβ2 mRNA)	100	Vinayagam [43]	Poor response to TAM in ERα^+^/PR^−^ BCa	115	Saji [59]
	Better TAM response, longer OS	74	Palmieri [44]	High-grade tumor and progression of BCa	53	Leygue [60]
	Better RFS and OS in patients with TAM treatment	105	Davies [45]	Carcinogenesis and invasive BCa	151	Esslimani-Sahla [61]
	Better outcome in tumors with higher ERβ2 than ERβ1 in late-onset patients	74	Mandusic [46]	Associated with lympho-vascular invasion	44	Bozkurt [62]
				High expression during growth and tumor progression of BCa	57	Omoto [63]
				ERβ exon5 splice variant mRNA in grade III tumors in postmenopausal women	40	Poola [64]
**ERβ5**	Improved survival with nuclear ERβ5 expression	850	Shaaban [41]	High ERβ3 and ERβ5 protein expression with large tumor and node-positive BCa	17	Chi [65]
	Better RFS with TAM treatment	105	Davies [45]	High-grade tumor and progression of BCa	53	Leygue [60]

RFS, relapse-free survival; DFS, disease-free survival; OS, overall survival; TAM, tamoxifen; AAW, African American women; TNBC, triple-negative breast cancer.

## Data Availability

The datasets generated and/or analyzed during the current study are included in this manuscript. Any additional data are available from the corresponding author on reasonable request.
